# Biological Effects of Polysaccharides from *Bovistella utriformis* as Cytotoxic, Antioxidant, and Antihyperglycemic Agents: In Vitro and In Vivo Studies

**DOI:** 10.3390/pharmaceutics17030335

**Published:** 2025-03-05

**Authors:** Aya Maaloul, Claudia Pérez Manríquez, Juan Decara, Manuel Marí-Beffa, Daniel Álvarez-Torres, Sofía Latorre Redoli, Borja Martínez-Albardonedo, Marisel Araya-Rojas, Víctor Fajardo, Roberto T. Abdala Díaz

**Affiliations:** 1 Department of Ecology and Geology, Faculty of Science, University of Málaga, E-29071 Málaga, Spain; maalouleya6@gmail.com; 2 Grice Hutchinson Experimental Centre, Institute of Blue Biotechnology and Development (IBYDA), University of Málaga, Lomas de San Julián, 29004 Málaga, Spain; beffa@uma.es (M.M.-B.); dani-alto@uma.es (D.Á.-T.); 3 Laboratory of Chemistry of Natural Products, Department of Botany, Faculty of Natural and Oceanographic Sciences, University of Concepción, Concepción PC 304000, Chile; claudiaperez@udec.cl; 4 Biomedical Research Institute of Malaga and Platform in Nanomedicine (IBIMA-BIONAND Platform), Mental Health Clinical Management Unit, Hospital Regional Universitario de Málaga, Av. de Carlos Haya 82, 29010 Málaga, Spain; juandecara@uma.es; 5 Department of Cell Biology, Genetics and Physiology, Faculty of Science, University of Málaga, E-29071 Málaga, Spain; sofia.latorreredoli@uma.es; 6 Biomedical Research Institute of Malaga and Platform in Nanomedicine (IBIMA-BIONAND Platform), E-29071 Málaga, Spain; 7 Département of Sciences and Natural Resources, Faculty of Sciences, University of Magallanes, Punta Arenas 6200011, Chile; borja.martinez@umag.cl (B.M.-A.); marisel.araya@umag.cl (M.A.-R.); victor.fajardo@umag.cl (V.F.)

**Keywords:** *Bovistella utriformis*, polysaccharides, biomass composition, antioxidant activity, cytotoxicity, selectivity index, zebrafish toxicity, antihyperglycemic potential, β-cell preservation

## Abstract

**Background/Objectives**: This study explores the bioactive potential of *Bovistella utriformis* biomass and its polysaccharides (PsBu) through comprehensive biochemical and bioactivity analyses, focusing on their antioxidant, cytotoxic, and antihyperglycemic properties. **Methods**: Elemental analysis determined the biomass’s chemical composition. Antioxidant activity was assessed using ABTS and DPPH assays. Monosaccharide composition was analyzed via gas chromatography-mass spectrometry (GC-MS). In vitro cytotoxicity assays were conducted on cancer and normal cell lines to determine IC_50_ values and selectivity indices (SI). Zebrafish embryo toxicity was evaluated for teratogenic effects, and an in vivo antihyperglycemic study was performed in diabetic rat models. **Results**: The biomass exhibited high carbon content (around 41%) and nitrogen levels, with a balanced C/N ratio nearing 5. Protein content exceeded 50%, alongside significant carbohydrate, fiber, and ash levels. Antioxidant assays revealed inhibition rates of approximately 89% (ABTS) and 64% (DPPH). GC-MS analysis identified glucose as the predominant sugar (>80%), followed by galactose and mannose. Additionally, HPLC detected a photoprotective compound, potentially a mycosporin-like amino acid. Cytotoxicity assays demonstrated PsBu’s selective activity against colon, lung, and melanoma cancer cell lines (IC_50_: 100–500 µg·mL^−1^), while effects on normal cell lines were lower (IC_50_ > 1300 µg·mL^−1^ for HaCaT, >2500 µg·mL^−1^ for HGF-1), with SI values approaching 27, supporting PsBu’s potential as a targeted anticancer agent. Zebrafish embryo assays yielded LC_50_ values ranging from 1.4 to 1.8 mg·mL^−1^. In vivo, PsBu reduced fasting blood glucose levels in hyperglycemic rats (approximately 210 mg·dL^−1^ vs. 230 mg·dL^−1^ in controls) and preserved pancreatic β-cell integrity (around 80% vs. 65% in controls). **Conclusions**: These findings suggest that *B. utriformis* biomass and PsBu exhibit strong antioxidant activity, selective cytotoxicity against cancer cells, and antihyperglycemic potential, making them promising candidates for further biomedical applications.

## 1. Introduction

Macrofungi are well-known for their diverse nutritional and therapeutic properties [1], attributed to the bioactive chemical substances they produce [2]. These fungi have demonstrated a wide range of benefits for human health, including anti-inflammatory, antimicrobial, antiviral, immunomodulatory, and antihyperglycemic effects [3].

Among the bioactive compounds in macrofungi, polysaccharides have demonstrated significant therapeutic effects by acting through key mechanisms, including immune modulation, antioxidant activity, and the regulation of metabolic pathways [4,5,6]. These polysaccharides interact with innate immune receptors, enhancing protective immune responses while exhibiting low toxicity, making them promising candidates for various clinical applications. Their bioactivity has paved the way for innovative activities, such as the development of vaccines, in which their immunoprotective properties can be harvested. Additionally, fungal polysaccharides have shown great promise in antidiabetic therapies, the formulation of probiotics, renoprotective agents, and dietary supplements aimed at preventing or managing neurodegenerative diseases, underscoring their potential in addressing a wide range of health conditions [7].

Puffballs are macromycetes characterized by their globose, fleshy fruiting bodies, which enclose spores that are released upon maturity [8]. One notable species, *Bovistella utriformis* (*B. utriformis*), belongs to the Basidiomycota phylum and *Lycoperdaceae* family [9]. This wild saprophytic fungus thrives in sandy and grassy terrains, fructifying in autumn and summer. While edible in its juvenile stage, *B. utriformis* has a light fungal aroma, firm texture, and white color that turns brown with maturation [10]. In Chile, it is rare and localized in the southern regions, but it is also found in Spain and other European countries [11].

It is noteworthy to highlight that, because of the metal bioaccumulation capacities *B. utriformis* have shown to possess, they should not be collected in polluted areas [12,13,14].

Studies have shown that *B. utriformis* contains bioactive compounds which have been used for medicinal purposes since antiquity [15]. Some of these are calvatic acid, an azoxyformonitril with antiviral [16], antibacterial, antifungal, antioxidant, and antitumor activity [17,18,19,20,21]; lovastatin, a cholesterol-reducing agent by inhibition of 3-hydroxy-3-methyl-glutaryl-coenzyme A reductase (HMG-CoA-R) [22]; and calcaelin, a protein with antitumor effects in breast cancer [23]. Furthermore, this species also has an inhibitory effect on tyrosinase, acetylcholinesterase, and butyrylcholinesterase enzymes [22], and has notable hemostatic properties [24]. In its mature stage, the fruiting bodies become a powder which has been used to heal wounds in traditional medicine. Their extract is rich in ergosterol, N-acetylglucosamine, and α-tocopherol [22].

In addition to these bioactive molecules, the biomass of fungi, particularly macrofungi, is known to encompass a variety of lipophilic compounds, including lipids, sterols, and triterpenoids, which contribute significantly to their biological activities. For instance, triterpenoids found in *Ganoderma lucidum* have been shown to possess a range of pharmacological effects, including anticancer and hepatoprotective properties, as well as antiatherogenic effects seen in the alleviation of oxidative stress and inflammation [25]. Additionally, sterols such as ergosterol, prevalent in many fungi, exhibit significant antioxidant and anti-inflammatory activities [26]. The *Pleurotus* species, particularly *P. ostreatus*, have also demonstrated notable antioxidant and anti-inflammatory properties, with studies indicating that extracts from these mushrooms can suppress nitric oxide production in inflammatory models [27]. Moreover, the relationship between lipophilicity and biological activity in antifungal agents has been explored, noting that compounds with higher lipophilicity often exhibit enhanced antifungal properties [28].

Given the bioactivity of fungal polysaccharides, research on their therapeutic potential has intensified. Based on this research, in the present study, the potential anticancer, antihyperglycemic, and antioxidant properties of the polysaccharides present in *B. utriformis* (PsBu) were evaluated.

In the last decade, several in vivo toxicity assays using zebrafish embryos have arisen as alternatives to in vivo toxicity models in rodents [29,30,31,32]. Rapidity, externality, numerous offspring, and optical transparency are relevant features of zebrafish development that facilitate high-throughput, and cost-effective drug and chemical screens [33,34,35,36,37]. Genetic similarity with humans also drives translational activities [33,38,39].

In this study, we characterized the biochemical composition of *Bovistella utriformis* biomass, assessed its antioxidant potential, and investigated the in vitro cytotoxic effects of its polysaccharides on various cancer cell lines, highlighting their selective activity and potential therapeutic applications. Additionally, we evaluated the growth, toxicity, and lethality effects of polysaccharide extracts from the *Bovistella* genus using a zebrafish embryo assay. These assays have previously been utilized to study the biological activities of algal polysaccharides [40]. Furthermore, we analyzed the in vivo safety of these polysaccharides and their hypoglycemic and antitoxic activities in an animal model of paracetamol-induced toxicity.

## 2. Materials and Methods

### 2.1. Chemicals and Reagents

The 2,2-diphenyl-1-picrylhydrazyl (DPPH),ethidium bromide and 3-(4,5-dimethyl-thiazol-2-yl)-2,5-diphenyltetrazolium bromide (MTT) were purchased from Himedia Laboratories Pvt. Ltd. (Mumbai, India). Dulbecco’s modified Eagle’s Medium (DMEM), Roswell Park Memorial Institute Medium (RPMI-1640), minimal essential media (MEM), fetal bovine serum (FBS), penicillin, streptomycin sulfate salt powder, glutaraldehyde, acridine orange, propidium iodide, 2,7-dichloro dihydro fluorescein diacetate (DCFH-DA), rhodamine 123 (Rh123), Hoechst 33342 and fluoromount were obtained from Sigma-Aldrich (St. Louis, MO, USA). The remaining chemicals and reagents used for experimental analysis were of analytical grade.

### 2.2. Sample Collection

**Figure 1:**
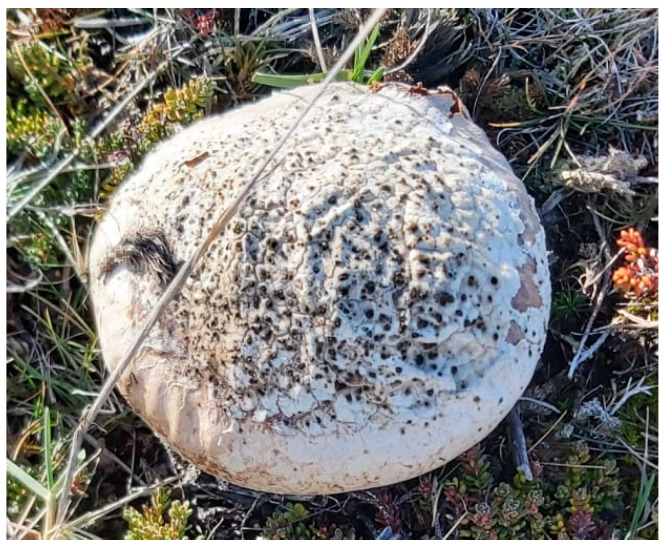
*Bovistella utriformis* fungus collected in its natural habitat for the study.

*B. utriformis* specimens (Figure 1) were collected in December 2022, in Tierra del Fuego (Chile) (53°32′14.2″ S y 068°42′10.3″ W) by Dr. Roberto Abdala Díaz and Dr. Víctor Fajardo. A specimen was identified based on its macroscopic characteristics and deposited in the mushroom collection of the University of Magallanes, Chile (0304UMAG voucher). Fresh carpophores were cleaned, chopped, and frozen (−20 °C) before lyophilization (Lyophilizer Cryodos, Telstar Lyo Quest, Málaga, Spain). The lyophilized biomass of *B. utriformis* specimens was used as the initial material for further analyses.

### 2.3. Total Carbon, Hydrogen, Nitrogen, and Sulfur Composition

The total carbon (C), hydrogen (H), nitrogen (N), and sulfur (S) composition in the lyophilized *B. utriformis* biomass was determined using the total combustion method with a LECO TruSpec Micro CHNSO elemental analyzer(St. Joseph, MI, USA), following the protocol described by Abdala Díaz, R [41]. The content of each element (C, H, N, S) was expressed as a percentage (%) with respect to the sample’s total weight. All determinations were made in triplicate (*n* = 3).

### 2.4. Biochemical Composition

To determine the biochemical composition, analyses were conducted on biomass that had been previously lyophilized. Lipid content was assessed following the Folch method [42], while total protein content was calculated by applying the Lourenço [43] N-prot factor of 4.99—specific to microalgae in the early stationary phase to the total nitrogen percentage. Carbohydrate levels were quantified using the Dubois method [44], and inorganic compound weight was measured using the approach described by Ismail [45]. The gravimetric analysis determined ash content by incinerating the samples in a muffle furnace at 550 °C for twelve hours. All experiments were performed independently and repeated three times (*n* = 3).

### 2.5. Determination of Total Phenolic Content

Total phenolic content in the lyophilized *B. utriformis* was calculated using Folin–Ciocalteu assay with few modifications and expressed as gallic acid equivalents (GAE) per gram of dry weight (DW) [46]. The absorbance was measured at 760 nm using a UV–visible spectrophotometer (SHIMADZU UV MINI-1240, Duisburg, Germany). The concentrations of phenolic contents were determined by constructing a standard curve using different concentrations of gallic acid (Sigma-Aldrich, St. Louis, MO, USA).

### 2.6. High-Performance Liquid Chromatography (HPLC)

The identification of mycosporine-like amino acids (MAAs) was performed using high-performance liquid chromatography (HPLC) with an Agilent-1260 HPLC system. A C8 column packed with 5 µm material, coupled with a pre-column, was used to enhance the separation of compounds.

The mobile phase consisted of 1.5% methanol and 0.5% acetic acid, mixed with distilled water and maintained without the presence of air. The elution flow rate was set to 0.5 mL/min, and a sample volume of 20 to 40 µL was injected for each analysis. The total elution time was 30 min. Scans were performed using a UV–Visible spectrum detector, covering wavelengths from 290 to 400 nm, with measurements taken every second to ensure accurate results [47].

### 2.7. Antioxidant Activity

#### 2.7.1. ABTS Radical Scavenging Activity

The ability of the fungus to scavenge the 2,2′-azino-bis (3-ethylbenzothiazoline-6-sulfonic acid (ABTS) radicals was evaluated using an ABTS assay, as reported by Re, R et al. [48], with a few modifications. The ABTS radical cation was generated by a reaction of 7 mM ABTS (Sigma-Aldrich, St. Louis, MO, USA) with 2.45 Mm potassium persulphate (Sigma-Aldrich, St. Louis, MO, USA). This reaction mixture was stored for 16 h at room temperature and used within two days. After incubation, the mixed solution was diluted to a 0.7 absorbance unit at 413 nm with the deionized water. From a lyophilized biomass weight of 10 mg, dissolved in 1 mL of phosphate buffer, dilutions were made so that the final concentrations in the cuvettes were 12.5, 25, 50, 100, and 200 µg mL^−1^. Then, 50 µL of these samples were mixed with 940 µL of phosphate buffer and 10 µL of ABTS solution in the cuvettes. Then, the mixture was measured at 413 nm. ABTS radical scavenging capacity was calculated according to the following equation:

AA% = [Abs (control) − Abs (sample)/Abs (control)] × 100

where Abs (control) is the absorbance of the ABTS radical in phosphate buffer at time 0; Abs (sample) is the absorbance of the ABTS radical solution mixed with the sample after 8 min. From a stock of Trolox 2.5 mM a calibration curve was made with different concentrations of 6-hydroxy-2,5,7,8-tetramethylchroman-2-carboxylic acid (Trolox). The serial dilutions were obtained at concentrations of 5, 10, 15, and 20 µM. All determinations were performed in triplicate (*n* = 3) [49]. The antioxidant capacity was expressed as percentage using the Trolox equation: y = 0.0043x + 0.0124, R^2^ = 0.9943.

#### 2.7.2. DPPH Radical Scavenging Activity

The antioxidant and radical scavenging activities of *B. utriformis* were evaluated by the DPPH free radical method of Brand-Willians et al. [50]. From a mushroom dry weight of 1 mg, an extract was prepared by adding 1 mL of 80% methanol (MeOH) to the sample. Dilutions were prepared to achieve final concentrations of 200, 100, 50, 25, and 12.5 µg mL^−1^ in the cuvettes. A solution of DPPH (Sigma-Aldrich, St. Louis, MO, USA) at 1000 µL was prepared with 80% methanol (MeOH) and combined with 200 µL of the sample in cuvettes. The initial absorbance was measured at 517 nm. Following a 30 min incubation period, absorbance was recorded again at the same wavelength, with 80% MeOH as the blank control. The absorbance values were subsequently converted into percentage inhibition relative to the control. The antioxidant activity percentage was calculated according to the following Equation:

AA% = [(Abs0 − Abs1)/Abs0] × 100

where Abs0 is the absorbance at time 0 min and Abs1 is the absorbance at the end of the reaction (30 min) at 517 nm. A calibration curve was established using various concentrations of Trolox, prepared from a stock solution of 1.268 mM. Dilutions were obtained at concentrations from 0 to 6 µM. All determinations were performed in triplicate (*n* = 3) [41]. The antioxidant capacity was expressed as percentage using the Trolox equation: y = 0.0043x + 0.0124, R² = 0.9943.

### 2.8. Extraction of Polysaccharides

The extraction of polysaccharides from *Bovistella utriformis* (PsBu) was conducted following the method described by [51]. The fungal biomass of *B. utriformis* was initially treated with absolute ethanol (EtOH) to remove pigments. This process was repeated three times until the ethanol became crystal clear. The biomass was subsequently centrifuged and resuspended in distilled water at a ratio of 1:10 (*w*/*v*). This mixture was then heated to boiling and maintained at that temperature for approximately 1 h with continuous stirring. Subsequently, the mixture was centrifuged at 4500 rpm and 4 °C for 10 min.

The supernatant was collected, and phenolic compounds were precipitated using 1 mg of polyvinylpolypyrrolidone (PVPP). After an additional centrifugation under the same conditions, the pellet was discarded. The resulting supernatant was cooled on ice to 10 °C, and polysaccharides were precipitated by adding cold absolute ethanol (*v*/*v*). The solution was left to stand until the formation of white flocculent precipitates occured, which were collected by centrifugation. The precipitated polysaccharides were subsequently frozen in absolute ethanol at −80 °C for 24 h and lyophilized using a Cryodos Lyophilizer (Telstar) (Lyophilizer Cryodos, Telstar Lyo Quest, Málaga, Spain).

### 2.9. Fourier Transform Infrared Spectroscopy

Fourier Transform Infrared Spectroscopy (FT-IR) analysis was used to characterize the structure of the PsBu and to identify the functional groups in the polysaccharide structure [52]. The FT-IR spectra in the 400–4000 cm^−1^ region were obtained using self-supporting pressed disks of 16 mm in diameter of a mixture of polysaccharides and potassium bromide (1% *w*/*w*) with a hydrostatic press at a force of 15.0 t cm^−2^ for 2 min. A Thermo Nicolet Avatar 360 IR spectrophotometer (Thermo Electron Inc., Franklin, MA, USA) with a resolution of 4 cm^−1^, with detectors for Fourier Transform Spectroscopy (DTGS), was operated with OMNIC 7.2 software (bandwidth 50 cm^−1^, enhancement factor 2.6). Baseline adjustment was performed using the Thermo Nicolet OMNIC software 9 (Termo Fischer, San Jose, CA, USA). The OMNIC correlation algorithm was used to compare sample spectra with those of the spectral library (Thermo Fischer Scientific).

### 2.10. Monosaccharide Composition Analysis

#### 2.10.1. Methanolysis and Derivatization

In a 3 mL reaction vial (Thermo), 2 mg of the extracted PsBu were subjected to acid methanolysis with 600 µL of 3 N HCl in MeOH (Sigma Aldrich) at 80 °C for 24 h. The solvent was then evaporated under a nitrogen stream at 50 °C using an evaporator-concentrator (Stuart Block Heater, SBH200D/3, Stafford, UK).

The residue was washed three times with MeOH to eliminate excess acid, and dried again. The samples were then derivatized via silylation with 300 µL of Tri-Sil reagent (Pierce, Thermo) at 80 °C for 1 h. After derivatization, the reagent was removed using a nitrogen stream, and the residue was reconstituted in 500 µL of hexane. The solution was centrifuged for 15 min, and the supernatant was filtered, evaporated once more, and reconstituted in 150 µL of hexane (LC-MS grade, Sigma) in a chromatography vial.

#### 2.10.2. Gas Chromatography/Mass Spectrometry Analysis

The PsBu fractions were characterized using Gas Chromatography-Mass Spectrometry (GC-MS) with an Agilent 7890A system (Santa Clara, CA, USA), with an Agilent 5975C mass detector, using an HP5-MS type fused silica capillary column of 30 m, 0.25 mm inner diameter, and 0.25 μm film thickness, with the following characteristics: temperature: 250 °C; detector (mass): 280 °C; furnace: initial 100 °C for 5 min, increasing by 8 °C min^−1^ up to 250 °C and maintained for 15 min [53]. The detector set in the scan mode ranged from *m*/*z* 50 to 500. The carrier gas flow (electronic degree helium) was at 1 mL min^−1^. The compound characterization was carried out by comparison with the NIST^®^ database.

### 2.11. Cytotoxic Effect Assay of Polysaccharides from B. utriformis

The cytotoxic effects of PsBu samples were evaluated using six human cell lines: HCT-116 (colon cancer), 1064Sk (fibroblasts), G-361 (melanoma), NCI-H460 (lung cancer), HGF1 (fibroblasts), and HaCaT (keratinocytes). Cells were cultured in either DMEM (Capricorn Scientific, ref. DMEM-HPSTA) or RPMI-1640 medium (BioWhittaker, ref. BE12-167F), both supplemented with 10% Fetal Bovine Serum (FBS, Biowest, ref. S1810-500), 1% penicillin-streptomycin solution 100× (Capricorn Scientific, ref. PS-B), and 0.5% amphotericin B (Biowest, ref. L0009-100). The cultures were maintained under sub-confluent conditions at 37 °C in humidified air containing 5% CO_2_.

The cytotoxic effect was measured using the MTT assay in the above-mentioned cell lines. Cells were seeded into 96-well plates and independently exposed to varying concentrations of the PsBu for 72 h. The trial was carried out following the method proposed in [41]. Cytotoxicity was expressed as the half-maximal inhibitory concentration (*IC*_50_) values. Additionally, the selectivity index (SI) was calculated to evaluate the specificity of the polysaccharides for cancerous versus non-cancerous cells.

$$SI=\frac{{IC}_{50}^{healthy\;cells}}{{IC}_{50}^{tumor\;cells}}$$

The analyses were carried out in three independent experiments.

### 2.12. In Vivo Study

#### 2.12.1. Zebrafish Embryo Husbandry

*Danio rerio* embryos are the offspring of a mating between wild-type AB adults cultured at standard conditions [54,55]. Adults were reared at the Centro de Experimentación y Conducta Animal (CECA) Universidad de Málaga, Spain. Prior to experiments, fertilized eggs were bleached in Petri dishes and acclimatized at 28 °C [56]. The Universidad de Málaga Bioethics Commission approved the experiments that were run under permission 92-2018-T and the grant UMA18-FEDERJA-274.

#### 2.12.2. Zebrafish Embryo Assay

Fifteen randomized 2–6 h post fertilization embryos, hpf, were placed in 3 mL embryo medium in 6-well plates following previous reports [57]. Embryos showing abnormal development were not included. For three days, embryos were immersed in different concentrations of PsBu.

PsBu concentrations were obtained by dilution of 5 mg mL^−1^ stock solutions in E3 medium [56]. Stock solutions were stored at 4 °C. Incubation in E3 embryo medium and 2 mg mL^−1^ *Sarcopeltis skottsbergii* PSE [58] were used as negative and positive controls, respectively. Experiments were run in triplicate in consecutive weeks. Embryo euthanasia was performed by over-anesthesia. Embryos were eliminated as organic waste (SEPRUMA-UMA).

#### 2.12.3. Phenotypic and Morphometric Analysis

Each day several phenotypic variables (i.e., viability, hatching, cardiac edema) were recorded under the optical microscope (Nikon SMZ-445 model, Tokyo, Japan). At 72 hpf, digital images were obtained using a Nikon Microphot-FX Fluorescence Microscope with a Nikon DS-L1 camera. When possible, annotated variables were confirmed based on digital images. Viability data were used to estimate LC_50_ using both a log–linear [59] and a log–log regression test [60]. The standard length (SL) of each specimen, measured using digital images by ImageJ 1.50i settings (National Institutes of Health, Bethesda, MD, USA), were used to estimate growth delay [54,61]. Differences between means of the standard length of embryos under different experimental conditions were statistically determined by *t*-test (Statgraphic, Statgraphic Technology, Inc., The Plains, VA, USA).

#### 2.12.4. Animal Protocol and Ethics Statement for Mice In Vivo Study

For the animal study, 40 C57BL/6 mice (Charles River, Barcelona) were provided and housed by CECA. All animals, weighing approximately 22 ± 3 g at the start of the study, were maintained under standard laboratory conditions, including housing in cages at an ambient temperature of 22–25 °C, 75% humidity, and a 12 h light/dark cycle. Water and balanced rodent feed were provided ad libitum. Handle and care of animals followed the European Communities Council Directives 2010/63/EU (Regulation EC 86/609/ECC, 24 November 1986) and Spanish National and Regional Guidelines for Animal Experimentation (Real Decreto 53/2013). As well, experimental animal protocols and procedures were approved on 31 May 2021, by the local ethical committee for animal research of the Universidad de Málaga (Ref. no. 43-2021-A), and performed following the ARRIVE guidelines (Animal Research: Reporting of In Vivo Experiments). All efforts were made to minimize animal suffering and reduce the number of mice used per experimental group.

#### 2.12.5. Oral Glucose Tolerance and Insulin Sensitivity Tests

Mice were fasted for 16 h before undergoing an oral glucose tolerance test (OGTT). The animals were administered a glucose overload of 2 g kg^−1^ body weight (BW) via gavage, in a 10 mL kg^−1^ BW volume. Thirty minutes before the glucose load, animals received 200 mg kg^−1^ BW PsBu in 0.9% NaCl as vehicle at a volume of 10 mL kg^−1^ BW, or only vehicle, for the control group (CONT), via gavage. Tail blood samples were collected at 0, 5, 10, 15, 45, 30, 60, and 120 min after glucose administration. Blood glucose levels were measured as described previously [62] using a commercial glucometer based on the glucose oxidase method (Accu-Chek, Roche Diagnostics, Rotkreuz, Switzerland).

For the insulin sensitivity test (IST), animals were also fasted for 16 h and then administered insulin intraperitoneally (ip) at a dose of 0.5 IU kg^−1^ BW in a volume of 10 mL kg^−1^ BW. Thirty min before insulin administration, animals received 200 mg kg^−1^ BW of PsBu in a volume of 1 mL/100 g BW, or vehicle via gavage. From this point, the same procedure as that for the OGTT was followed, with tail blood samples collected at 0, 5, 10, 15, 30, 45, 60, 90, and 120 min after insulin administration, and blood insulin levels were measured.

#### 2.12.6. Chronic Treatments

The paracetamol (APAP, cat# A7085, Sigma-Aldrich, Saint Louis, MO, USA) solution (30 mg mL^−1^) for animal administration was prepared fresh daily by dissolving it in a vehicle containing 0.5% methylcellulose (cat# 428430500, Thermo Scientific, Waltham, MA, USA) in 0.9% NaCl. The PsBu solution also was prepared daily by weighing 60 mg dissolved in 3 mL of vehicle. For chronic treatment, the animals were divided into three groups of eight mice each (*n* = 8): the CONT group, which received only the vehicle; the APAP group, which received a dose of 300 mg kg^−1^ BW of APAP; and the PsBu group, which received 300 mg kg^−1^ BW of APAP along with 200 mg kg^−1^ BW of PsBu. All treatments were administered via gavage in a volume of 10 mL kg^−1^ BW for 40 days.

#### 2.12.7. Biochemical Analysis in Plasma

After the chronic treatment period, animals were anesthetized with an intraperitoneal overdose of sodium pentobarbital (50 mg kg^−1^ BW) and sacrificed 2 h after the last dose. Blood samples were collected in EDTA-2Na tubes and centrifuged at 2000× *g* for 10 min at 4 °C to obtain plasma, which was stored at −80 °C until biochemical analyses. The following plasma metabolites were then measured: glucose, triglycerides, total cholesterol, high-density lipoproteins (HDL), uric acid, urea, creatinine, bilirubin, and the liver enzymes aspartate aminotransferase (AST) and alanine aminotransferase (ALT). These metabolites were analyzed using a Hitachi 737 Automatic Analyzer (Hitachi Ltd., Tokyo, Japan).

### 2.13. Statistical Analysis

Data were expressed as the mean ± standard error of the mean (SEM) of at least 8 determinations per experimental group. Statistical differences between the means were calculated using the Student *t*-test for comparisons between the two groups. Linear regressions were performed using a least squares analysis. Differences were analyzed via a one-way or two-way ANOVA depending on the factors and type of analysis, followed by the Bonferroni post hoc test for multiple comparisons. Significant differences were noted when *p* < 0.05, *p* < 0.01, and *p* < 0.001. A *p*-value below 0.05 was considered statistically significant.

The statistical analyses were performed using the Statistical Package for the Social Sciences software (IBM, SPSS version 25), Statgraphics software (Statgraphics centurion 18 Technologies, Inc., The Plains, VA, USA), or Microsoft Excel (Microsoft Office, Windows 11, Redmond, WA, USA). Statistical results were obtained using GraphPad Prism version 6.01 (GraphPad Software Inc., San Diego, CA, USA).

## 3. Results

### 3.1. Chemical Assessment: Elemental Composition and Bioactive Potential of B. utriformis Biomass

The elemental analysis of B. utriformis biomass revealed a chemical composition dominated by carbon, with notable proportions of hydrogen, nitrogen, and minor sulfur content. The calculated molar C/N ratio of 4.86 indicates a balanced carbon and nitrogen profile, which is essential for evaluating the biomass’s biochemical potential.

The dry weight composition analysis demonstrated a predominance of proteins, accompanied by substantial levels of carbohydrates and fibers, with smaller contributions from lipids and ash.

Monosaccharide profiling confirmed glucose as the principal sugar, with lesser amounts of galactose and mannose, reflecting the polysaccharide composition of the biomass. Additionally, the biomass contained a measurable concentration of total phenolic compounds, which correlated with significant antioxidant activity, as demonstrated by the ABTS and DPPH assays. These results underscore the bioactive potential of B. utriformis as a source of antioxidants and nutritional components.

Furthermore, the polysaccharide extraction yield was determined to be 9.56 ± 0.12%, indicating a substantial recovery of bioactive macromolecules (Table 1).

### 3.2. High Performance Liquid Chromatography

**Figure 2:**
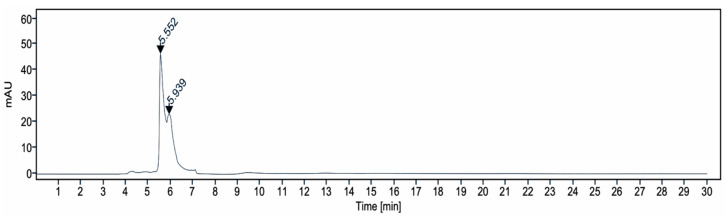
Chromatogram obtained by HPLC of *B. utriformis*.

**Figure 3:**
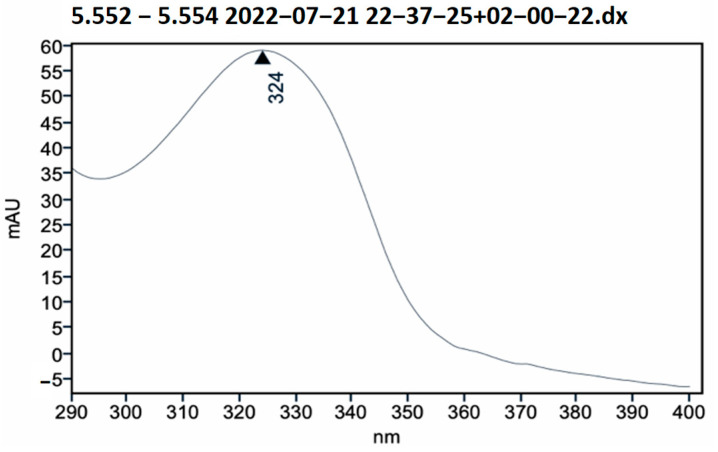
HPLC absorption spectrum of *B. utriformis*.

High performance liquid chromatography (HPLC) (Figure 2 and Figure 3) was used to analyze the *B. utriformis* biomass. A notable peak was observed at 324 nm, suggesting the presence of a photoprotective compound at this wavelength. This compound could be an mycosporin-like amino acid, which is known for its photoprotective properties.

### 3.3. Fourier Transform Infrared Spectroscopy

**Figure 4:**
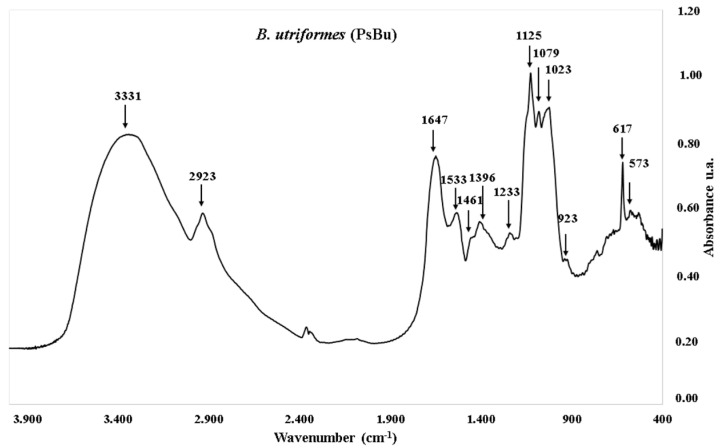
Fourier transform infrared spectroscopy (FT-IR) of polysaccharides obtained from *B. utriformis* polysaccharides (PsBu).

The FT-IR spectrum of PsBu displayed several characteristic absorption bands indicating the main functional groups present (Figure 4). The broad peak at 3331 cm^−1^ corresponds to O–H stretching vibrations, and a band at 2923 cm^−1^ indicates C–H stretching vibrations. A relatively strong absorption peak appears at 1647 cm^−1^, likely associated with C=O bonds, while the band at 1533 cm^−1^ may indicate C=O or COO– stretching. Additional peaks include a band at 1461 cm^−1^, possibly due to C–H bending vibrations, and peaks at 1079 cm^−1^ and 1023 cm^−1^, associated with C–O–C glycosidic linkages in polysaccharides. Absorption bands within the range of 617 cm^−1^ to 573 cm^−1^ may reflect β-glycosidic linkages or other ring deformations in the structure of PsBu.

### 3.4. Qualitative Analysis of Samples by Gas Chromatography Coupled to Mass Spectrometry

**Figure 5:**
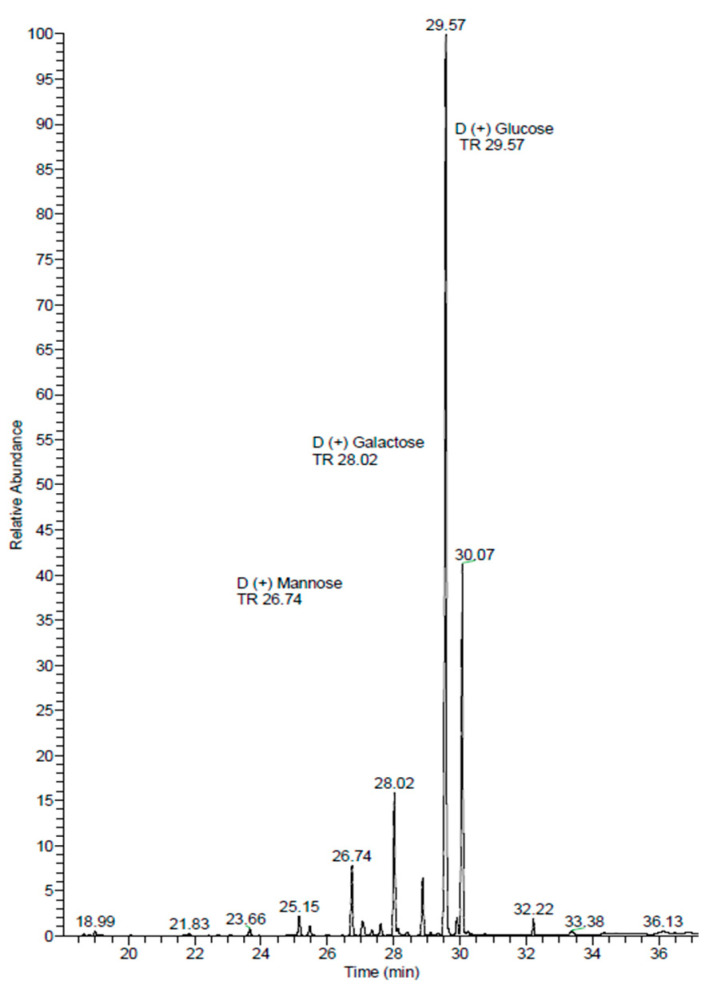
Qualitative analysis of Ps-Bu by gas chromatography coupled to mass spectrometry.

In this section, we present a comprehensive qualitative analysis of the monosaccharides extracted from the polysaccharides of *B. utriformis* using gas chromatography coupled with mass spectrometry (GC-MS). Following the analysis of retention times and mass spectra of monosaccharide standards, we successfully identified several monosaccharides, specifically glucose, mannose, and galactose, as detailed in Table 2 and Figure 5.

The quantitative assessment of these monosaccharides revealed that glucose was the predominant component, constituting approximately 82.76% of the total area under the chromatogram. This finding aligns with previous studies that have indicated glucose as a major constituent in various polysaccharide sources [63,64]. Galactose accounted for 11.92%, while mannose represented 5.32% of the total identified components. The cumulative area of all identified monosaccharides was 1,566,603,649.16, indicating a significant presence of these sugars in the polysaccharide matrix.

To further elucidate the structural diversity of the identified monosaccharides, we note that the analysis revealed multiple isomers for each sugar type. Specifically, two components were identified as mannose, three as galactose, and three as glucose. The retention times for these components are as follows: mannose at 26.74 min and 27.61 min; galactose at 28.02 min, 28.88 min, and 29.12 min; and glucose at 29.57 min, 30.07 min, and 32.22 min.

The observed retention time differences suggest the presence of isomeric forms, which could include α- and β-anomers, as well as linkage isomers. For instance, the two mannose components may correspond to α-mannose and β-mannose, differing in the orientation of the hydroxyl group at the anomeric carbon (C1) [65]. Similarly, the three galactose components may represent different stereoisomers or regioisomers, which can exhibit distinct biological activities [66]. The glucose components may also include α-D-glucose and β-D-glucose, along with potential linkage isomers, further complicating the analysis [67].

Understanding the structural diversity of these monosaccharides is crucial, as it can significantly influence their functional roles in biological systems and their interactions with other biomolecules [68]. The identification of these isomers highlights the importance of employing advanced analytical techniques, such as GC-MS, to achieve a detailed characterization of complex carbohydrate mixtures [69].

### 3.5. In Vitro Study

#### Cytotoxic Assessment of PsBu on Tumor and Healthy Cell Lines

**Figure 6:**
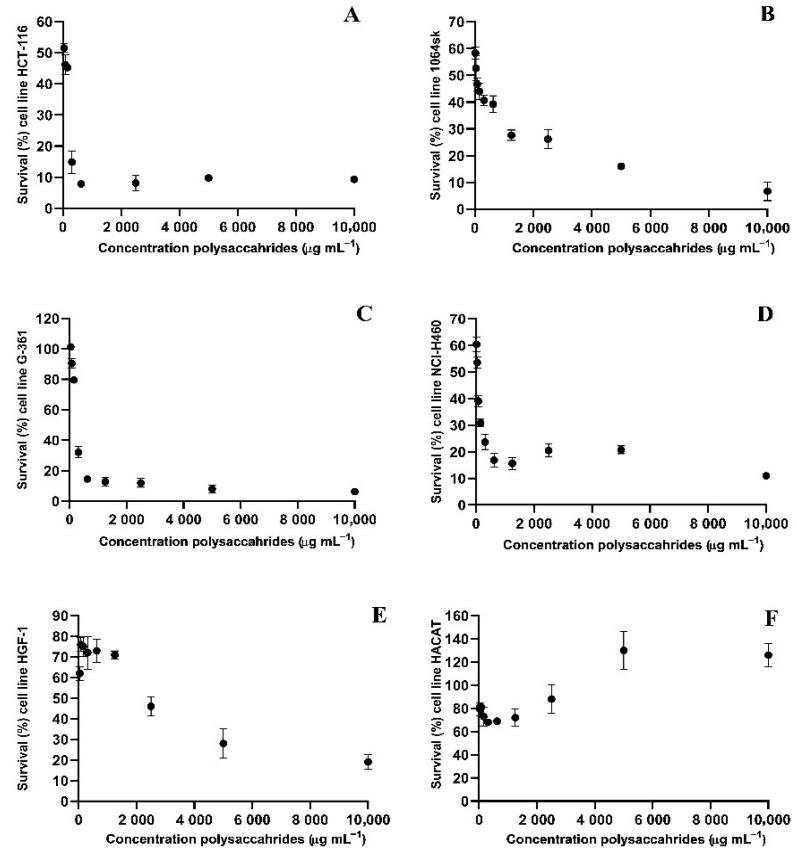
Survival rate of cell lines treated with PsBu. (**A**) HCT-116 (Colon Cancer), (**B**) 1064Sk (Fibroblasts), (**C**) G-361 (Melanoma), (**D**) NCI-H460 (Lung Cancer), (**E**) HGF-1 (Gingival Fibroblasts), and (**F**) HaCaT (Keratinocytes).

The cytotoxic effect of the PsBu was evaluated across various cell lines, revealing significant differences in survival rates and IC_50_ values among lung cancer (NCI-H460), colon cancer (HCT-116), and melanoma (G-361) cell lines, as well as in healthy human fibroblasts (HGF-1 and 1064SK) and keratinocytes (HaCaT). In the 1064SK fibroblast cell line, a minimal survival rate of 6.7% was observed at 10,000 µg mL^−1^, followed by 15.99% at 5000 µg mL^−1^, with an IC_50_ value of 117.58 µg mL^−1^, indicating a cytotoxic effect on these healthy cells. The HCT-116 colon cancer cell line exhibited the lowest survival rate of 7.80% at 1250 µg mL^−1^, with an IC_50_ value of 133 µg mL^−1^, demonstrating notable sensitivity toBuPs. For the NCI-H460 lung cancer cell line, the minimum survival rate was 10.93% at 20,000 µg mL^−1^ and 15.59% at 2500 µg mL^−1^, with an IC_50_ of 96.8 µg mL^−1^, highlighting its potent cytotoxicity. In the G-361 melanoma cell line, a minimal survival rate of 6.38% was recorded at the highest tested concentration, followed by 7.96% at the next highest concentration, and stabilization around 12% at concentrations as low as 1250 µg mL^−1^. The IC_50_ for this cell line was determined to be 500.5 µg mL^−1^. Interestingly, the HaCaT keratinocyte cell line, a model for healthy cells, did not exhibit significant cytotoxic effects. Instead, increased cell proliferation was observed at higher concentrations, suggesting potential beneficial effects of the BuPs on keratinocyte health rather than cytotoxicity with IC_50_ = 1319.28 µg mL^−1^. Similarly, the HGF-1 cell line exhibited low cytotoxicity, with the strongest effect observed at the highest concentration tested. The IC_50_ for HGF-1 was calculated as 2600.66 µg mL^−1^, indicating the limited cytotoxic impact on this cell line (Figure 6).

The selectivity index has also been obtained, with the following results (Table 3).

### 3.6. In Vivo Toxicity Study

#### 3.6.1. Zebrafish Lethality and Teratogenesis

**Figure 7:**
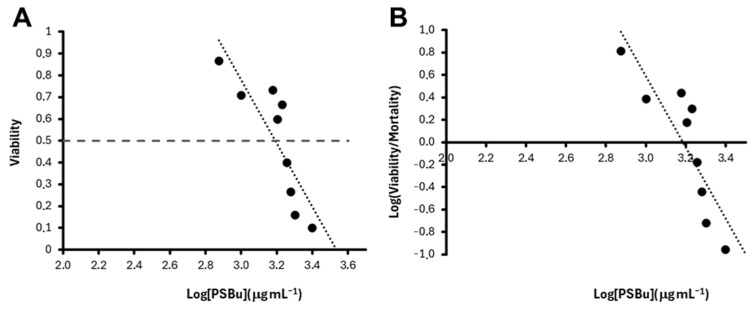
LC_50_ estimation following PsBu treatment of zebrafish embryos during 48 hpf. Plots show the linear relationship between viability (**A**) or the logarithm of the viability/mortality ratio (**B**) and the logarithm of concentration at 48 hpf. Intersections of regression lines with 0,5 viability index (**A**) and abscissa (**B**) are log (LC_50_) estimations. Linear adjustments are y = −1.591x + 5.6606 (R^2^ = 0.6511; *p* ≈ 0.0000) (**A**) and y = −3.1702x + 10.0968 (R2 = 0.7422; *p* ≈ 0.0000) (**B**) (Excel, Microsoft Office). LC50 values are in the text.

The increase in PsBu concentrations reduces embryo viability at 24, 48, and 72 hpf. At 72 hpf, for instance, the viability declines to zero between 750 and 2500 μg mL^−1^ (Figure 7) and LC_50_ can be measured using either a log-linear (Figure 7A) or a log-log-linear (Figure 7B) regression method. Control 2000 μg mL^−1^ *S. skottsbergii* PS neutral fraction reduced the embryo viability below 50% [58].

The results obtained following both methods (Figure 6) are similar, and the same is found at 24 or 48 hpf (Table 4). LC_50_ ranges between 1391 μg mL^−1^ (72 hpf, log-log) and 1825 μg mL^−1^ (24 hpf, log). All *p* values of these measurements are well below 0.001 (Table 4).

**Figure 8:**
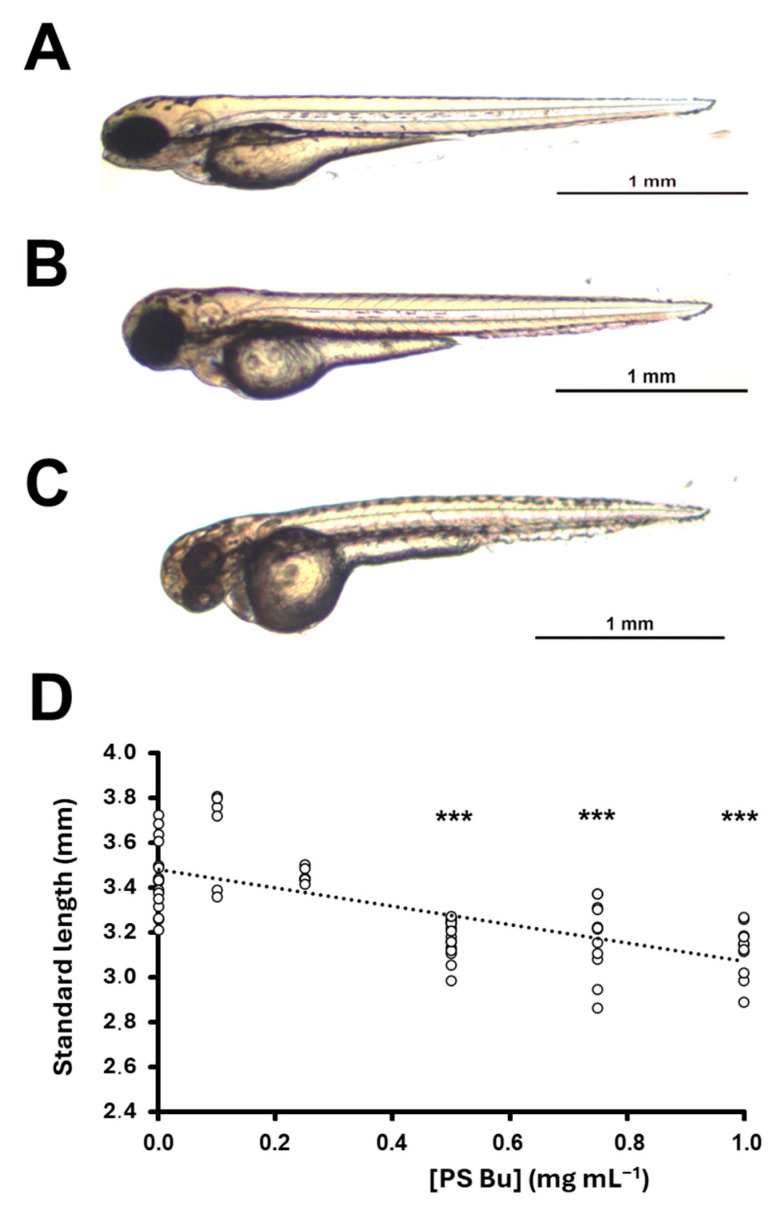
Body size reduction of 48 hpf zebrafish embryos induced by PsBu. (**A**–**C**). General morphology of E3 medium control (**A**) and PsBu-treated (**B**,**C**) larvae. Treating solutions were 0.75 (**B**) and (**C**) 1 mg mL^−1^ PsBu in the E3 medium. (**D**). Linear regressions of standard length (empty circles) with PsBu concentration (y = −0.4x + 3.4809; R^2^ = 0.4809; *p* ≈ 0.000). *** means comparison with E3 control data (t-student *p* < 0.001).

At intermediate concentrations, a slight delay in the growth of the embryo was observed (Figure 8A–D). We measured the standard length of 72 hpf larvae under several of these concentrations and plotted the values against PsBu concentrations. A linear reduction with a low R2 (0.4809; *p* ≈ 0.000; Figure 8D) and a slope of 0.4 mm mL^−1^ mL was obtained. If the *t*-test is calculated between experimental and negative control groups, only embryos in 0.5, 0.75, and 1 mg mL^−1^ PsBu groups show SL significantly different to the E3 medium group (Figure 8D). As with embryo viability, the control with a 2 mg mL^−1^ *S. skottsbergii* neutral fraction significantly reduced the standard length of embryos [58].

**Figure 9:**
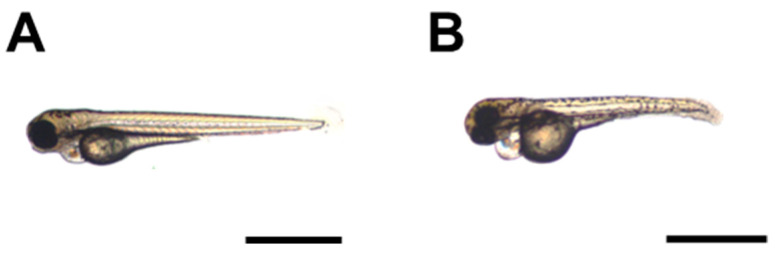
Teratogenic and toxic effects of PsBu over zebrafish embryos treated for 48 h. (**A**). Pericardial edema shown after 0.5 mg mL^−1^ PsBu treatment. (**B**). Size reduction, slight kyphosis and pericardial edema shown after 0.5 mg mL^−1^ PsBu treatment. Bar represents 1 mm.

Finally, we have also annotated other teratogenic effects such as kyphosis (Figure 9A) or pericardial edema (Figure 9A,B). Our data do not provide statistical significance to a potential relationship between these phenotypes and PsBu concentrations, although they are apparently more frequent at the higher concentration.

#### 3.6.2. Acute Administration of PsBu Improves Glucose Tolerance and Insulin Sensitivity in Mice

**Figure 10:**
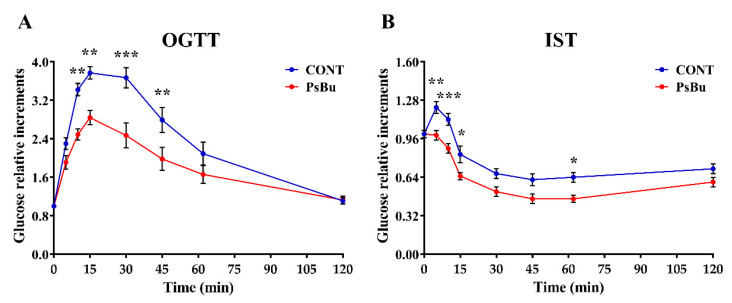
Glucose tolerance and insulin sensitivity. The effect of acute treatment with polysaccharides extracted from *B. utriformis* (PsBu) (200 mg kg^−1^) on the oral glucose tolerance test (OGTT) (**A**) and insulin sensitivity test (IST) (**B**) in male Wistar rats. Blood glucose levels were evaluated before (0 min) and after (0, 5, 10, 15, 30, 45, 60, and 120 min) glucose overload (2 mg kg^−1^) or insulin administration (1 IU kg^−1^). Points indicate the mean ± SEM (n ± 8 animals/group). Two-way ANOVA and Bonferroni post hoc test results were denoted as follows: (*) *p* < 0.05, (**) *p* < 0.01, and (***) *p* < 0.001 vs. vehicle group.

Since there is evidence that mushroom polysaccharides may have potential anti-diabetic effects [70], we evaluated the impact of acute PsBu treatment on glucose tolerance following a parenteral glucose load and on insulin sensitivity after acute insulin administration. In the OGTT, PsBu significantly improved glucose tolerance compared to control-treated mice, with pronounced effects observed at 10, 15, 30, and 45 min after the glucose load (Figure 10A). Similarly, in the ITT, PsBu-treated mice exhibited a marked reduction in blood glucose levels from 5 to 60 min following insulin injection, indicating a significant difference in insulin response between the control and treatment groups (Figure 10B).

#### 3.6.3. Chronic PsBu Administration Showed No Signs of Homeostasis Changes or Kidney and Liver Toxicities

Several biochemical parameters of blood plasma were evaluated after the experimental in vivo treatments, as shown in Table 5. The plasma levels of energy metabolism metabolites, such as glucose, triglycerides, cholesterol, and HDL, remained unchanged following chronic PsBu treatment compared to the control group. However, the administration of chronic APAP induced hyperglycemia, in comparison to both the control and PsBu-treated group, which did not. To verify that PsBu does not affect normal metabolite levels related to kidney and liver function, we measured uric acid, urea, creatinine, bilirubin, AST, and ALT. In this regard, no significant variations in renal damage markers or transaminases were detected in response to the PsBu with none of the treatments, except for bilirubin, which was elevated with both APAP and PsBu.

## 4. Discussion

The composition analysis of *B. utriformis* provides interesting results, particularly regarding its protein content. This mushroom exhibits an exceptionally high protein concentration of 52.80%, significantly exceeding the average expected for mushrooms, which is around 27.5%, according to Morais et al. [71]. However, this value aligns with the findings of Chandran, K et al. [58], who reported protein content of 42.98% in *Pleurotus florida*, highlighting that some mushroom species can exhibit remarkably high protein concentrations. These findings suggest that *B. utriformis* could possess considerable nutritional value in the food market due to its high protein content.

The high protein content of *B. utriformis* is directly correlated with its nitrogen concentration, as shown in its elemental analysis, which reveals a nitrogen content of 8.46%, much higher than the 3.95% reported by Zhang, J et al. [72]. Additionally, the presence of sulfur at a concentration of 0.59% may indicate enhanced biological activity.

In terms of carbohydrate content, *B. utriformis* has a percentage of 16.60%, which is considerably lower than the average reported by Morais et al. [71], but higher than that found in *Pleurotus florida* (11.34%) by Chandranet et al. [58]. The fiber content, calculated by difference, is 21.69%, significantly higher than that of *P. florida* (4.55%). It is important to note that these measurements were obtained from freeze-dried samples.

Regarding total phenolic compounds, *B. utriformis* shows a concentration of 6.23 ± 0.35 mgGAE/g. This value is consistent with those reported by Bach, F et al. [73], whose values range between 5.66 and 13.16 mg GAE.

The antioxidant activity of *B. utriformis* was assessed using both the ABTS and DPPH assays. The antioxidant activity measured by the ABTS method (88.96 ± 1.78%) was significantly higher than that obtained by the DPPH method (64.24 ± 2.75%). This discrepancy may be due to the exclusion of polysaccharides in the DPPH assay, which could lower the apparent antioxidant capacity of the mushroom. These results are comparable to those reported by [74], who observed variable antioxidant activity depending on the species and the methods used. For instance, in the ABTS assay, *Amanita caesarea* exhibited 92% antioxidant activity, while Boletus edulis showed 85.8%. In the DPPH assay, the results for *B. utriformis* are like those for *Agaricus bisporus* (67.86%) and *Pleurotus ostreatus* (86.35%).

The HPLC analysis of *B. utriformis* showed an irregularity in the spectrum at a wavelength of 324 nm, suggesting the presence of a mycosporine-like amino acid (MAA) with photoprotective properties. According to Alvarez-Gómez et al. [75] and Chaves-Peña et al. [76], similar compounds such as palythine and palythine-serine have been observed in various species at this wavelength.

The qualitative analysis via gas chromatography–mass spectrometry revealed the presence of several monosaccharides, including Glc, Man, and Gal. These findings partially align with those of Albornoz et al. [77], who identified Glc and Xyl in *Nothophellinus andinopatagonicus*.

The FTIR spectroscopy analysis revealed the presence of the hydroxyl groups indicated by the O–H stretching band at 3300.28 cm^−1^. This suggests that these functional groups may play an important role in the solubility and reactivity of PsBu, which is significant in biological contexts [78]. The C–H stretching at 2919.34 cm^−1^ points to aliphatic structures, which could contribute to hydrophobic interactions in the polysaccharide matrix. The observed C=O stretching at 1648.90 cm^−1^ and COO– stretching at 1564.53 cm^−1^ suggest the potential presence of uronic acid groups, which are known to enhance the biological activity of polysaccharides [79].

The C–O–C glycosidic linkages at 1099.27 cm^−1^ and 1015.38 cm^−1^ indicate a likely pyranose ring structure, which has been associated with enhanced immunomodulatory effects [80]. Finally, the absorption bands within 634.01 cm^−1^ to 570.63 cm^−1^ could suggest β-glycosidic linkages or other ring deformations, adding to the structural confirmation of PsBu [81]. These combined structural characteristics suggest potential applications for PsBu in anticancer and antioxidant treatments, given their association with beneficial biological activities.

In terms of cytotoxicity, the results of this study indicate that the polysaccharides extracted from *Bovistella utriformis* (PsBu) possess notable activity against several tumor cell lines, including NCI-H460 (lung cancer), HCT-116 (colon cancer), and G-361 (melanoma). The most pronounced efficacy was observed for NCI-H460, with an IC_50_ of 96.8 µg·mL^−1^, followed by HCT-116 (IC_50_ = 133 µg·mL^−1^) and G-361 (IC_50_ = 500.5 µg·mL^−1^). These observations align with previous studies demonstrating the antitumor activity of fungal polysaccharides [82].

Regarding healthy cells, 1064SK fibroblasts showed increased sensitivity with an IC_50_ of 117.58 µg·mL^−1^, while HGF1 and HaCaT cells exhibited lower sensitivity, with respective IC_50_ values of 2600.66 µg·mL^−1^ and 1319.28 µg·mL^−1^. Enhanced proliferation of HaCaT keratinocytes was observed at higher concentrations, suggesting that these polysaccharides may exert potential beneficial effects on these cells. This observation is consistent with research indicating that some fungal polysaccharides can positively modulate cell proliferation and immune activity [83].

The analysis of selectivity indices (SI) revealed a notable distinction between tumor lines and healthy cells. For NCI-H460, an SI of 13.63 relative to HaCaT and 26.87 relative to HGF-1 was observed, indicating favorable selectivity for this tumor line. Similarly, HCT-116 exhibited SIs of 9.92 and 19.55, respectively, while G-361 displayed lower indices (2.64 and 5.19). These results suggest that the efficacy of PsBu may vary depending on the tumor type, underscoring the need for specific evaluations to determine their therapeutic potential [84].

Despite these promising results, further studies are essential to elucidate the mechanisms underlying the cytotoxic activity of *B. utriformis* polysaccharides. Molecular analyses should explore their capacity to induce apoptosis or disrupt the cell cycle in tumor cells. Additionally, the toxicity observed in 1064SK fibroblasts highlights the importance of developing strategies to minimize adverse effects on healthy cells. Approaches such as encapsulating polysaccharides in nanoparticles could improve targeting while reducing systemic toxicity [85].

We have further tested the cytotoxic effect on cancer cells in a zebrafish embryo assay. A previous study [86] has stressed molecular similarities between zebrafish embryogenesis and human cancer. This study suggests that zebrafish embryo may be used as a proper screening assay to evaluate potential anticancer compounds. Other studies [86,87,88] have also evaluated the toxicity of algal and fungi polysaccharides over zebrafish embryos. We have followed these hypotheses and searched for the LC_50_ and teratogenic effects over the early developing zebrafish.

Although some polysaccharide extracts from algae do not show a measurable LC_50_ [86], others from either algae or fungi induce a significant lethality during zebrafish embryogenesis [86,87,88]. *Fucus vesiculosus* alginate [86], *Ganoderma applanatum* exo- or endopolysaccharides [88], *Lignosus rhinoceros* natural mycelial biomass or exopolysaccharides [87], and *Ulva rigida* PSE [61] show LC_50_ values that range between 245 μg mL^−1^ after 24 h (*Fucus vesiculosus*) and 5000 μg mL^−1^ after 48 (*Ulva rigida*) or 72 h (*Lignosus rhinoceros*). The LC_50_ observed in our study suggests an intermediate in vivo toxicity of PsBu, lower than that observed for *Fucus vesiculosus* alginate [86] but higher than the rest.

In this sense, zebrafish depigmentation induced by polysaccharide concentrations have been molecularly understood as an interference of tyrosine kinase and thus related with carcinogenesis inhibitors [75,89]. Moreover, PsBu depigment the embryos, although it also slightly reduces the size in a gradual linear manner suggesting an effect on developmental progression. This has been previously observed in zebrafish in vivo assay testing PSE from *Ulva rigida* [86,90] or *Sarcopeltis skottsbergii* in neutral extracts [58]. Other notorious effects at high concentrations are kyphosis or lordosis. Part of the phenotypic panel of the zebrafish embryo acute toxicity test (ZET) or the General and Behavioral Embryo Toxicity Assay [91] include these effects, and some of them have been related to carcinogenesis [85]. According to this explanation, the observed phenotypes are compatible with the above-mentioned in vitro anticancer activities. Pericardial edema was also observed at high concentrations, previously proposed to be associated with toxicity [55]. In general, these effects were also observed after the incubation of zebrafish embryos in both ulvan [59] and *Sarcopeltis skottsbergii* neutral [58] PSE solutions, suggesting a conserved effect of polysaccharides administered to zebrafish embryos

Promoting effects of zebrafish tail fin regeneration at very low concentrations have been found for *Spirulina maxima* pectins [40]. We are in the process of studying these effects after administration of *B. utriformis* in a similar regeneration assay. These doses are in the range of the cytotoxic IC50 indexes [92,93].

Acute PsBu treatment improves glucose handling and increases insulin sensitivity, suggesting increased insulin signaling in C67BL/6 mice. There is clear evidence that polysaccharides from some species of mushrooms could be capable of modulating the insulin signaling pathway to exhibit hypoglycemic and hypolipidemic effects and modulating the composition of gut microflora [94]. Previous studies in diabetic animal models showed that polysaccharides extracted from several mushrooms such as *Grifola frondosa* and *Pleurotus eryngii* can have the capability to regulate mRNA levels of key proteins involved in the insulin signaling pathways such as insulin receptor substrate (IRS) and kinases involved in the insulin signaling pathway [95,96,97]. Currently, the study of mushroom polysaccharides with potential therapeutic effects against insulin resistance and as modulators of the intestinal microbiota is yielding hopeful results [59]. The high prevalence of diseases associated with insulin resistance, such as type 2 diabetes, is well-documented in developed countries. Findings like these expand the range of therapeutic alternatives, particularly through functional foods and nutraceuticals, to address these conditions and their complications.

The analysis of plasma biomarkers revealed a safe pharmacological profile for chronic oral administration of PsBu in C57BL/6 mice, showing no significant impact on glucose homeostasis, lipid levels, renal function, or liver function. These findings strongly support the idea that the potentially beneficial chronic effects of PsBu are likely to be free of significant side effects. Consequently, PsBu could be added to the growing list of safe edible and medicinal mushrooms studied by polysaccharides for their therapeutic properties [98].

## 5. Conclusions

These findings indicate that the polysaccharides derived from *Bovistella utriformis* exhibit significant potential as an antioxidant, anticancer, and antihyperglycemic agent, suggesting their applicability in cancer therapy, diabetes management, and photoprotection. The selective cytotoxicity observed against cancer cell lines, with a high selectivity index, highlights their potential as targeted therapeutic agents while minimizing adverse effects on healthy cells. Additionally, their ability to lower blood glucose levels and enhance pancreatic β-cell preservation underscores their relevance in metabolic disorder management. Moreover, the biochemical characterization revealed a monosaccharide composition dominated by glucose, which may be linked to their bioactivity. The detection of a photoprotective compound, potentially a mycosporin-like amino acid, further expands their possible applications in dermatology and UV protection. However, the observed in vivo toxicity in zebrafish, with teratogenic and lethal effects at higher concentrations, underscores the necessity for comprehensive toxicological and pharmacokinetic studies to establish safe and effective dosage regimens. In summary, this study provides strong promise for future advancements in biomedical and pharmaceutical applications.

## Figures and Tables

**Table 1:** Elemental composition, dry weight components, monosaccharide composition and antioxidant activity of *B. utriformis* biomass.

Elemental Composition									
% C		% H			% N		% S		
41.143		6.182			8.461		0.597		
Content of proteins, carbohydrates, lipids, inorganic compounds, and fiber (% of BU).									
Ash		Proteins		Lipids		Carbohydrates		Fibers	
5.84		52.80		3.07		16.60		21.69	
Determination of total phenolic compounds and antioxidant activity									
Total phenolic compounds (mg eq AG/g DW)			ABTS			DPPH			
6.23 ± 0.35			88.96 ± 1.78%			64.24 ± 2.75%			

**Table 2:** Monosaccharides detected in PsBu.

Monosaccharide	TR (min)	Area
Mannose (Man)	26.74	71,179,113.99
Mannose	27.61	12,222,878.14
Galactose (Gal)	28.02	130,042,008.5
Galactose	28.88	53,704,735.4
Galactose	29.12	2,965,543.129
Glucose (Glc)	29.57	949,340,432.8
Glucose	30.07	332,589,869.8
Glucose	32.22	14,559,067.4

**Table 3:** Selectivity index (SI) of PsBu for cancer cell lines compared to healthy cells (HaCaT, 1064SK and HGF1).

Cancer Cell Line	IC_50_ (µg mL^−1^)	Reference Healthy Cell Model	IC_50_ Healthy (µg mL^−1^)	Selectivity Index (SI)
HCT-116 (Colon)	133	HaCaT	1319.28	9.92
		HGF1	2600.66	19.55
		1064Sk	117.58	0.88
NCI-H460 (Lung)	96.8	HaCaT	1319.28	13.63
		HGF1	2600.66	26.87
		1064Sk	117.58	1.21
G-361 (Melanoma)	500.5	HaCaT	1319.28	2.64
		HGF1	2600.66	5.19
		1064Sk	117.58	0.23

**Table 4:** LC_50_ (μg mL^−1^) of PsBu over zebrafish embryos.

Age (hpf)	Log Regression		Log-Log Regression	
	LC_50_	*p* Value	LC_50_	*p* Value
24	1825	2.9536 × $\;10^{-8}$	1807	4.074 × $10^{-6}$
48	1553	2.3603 × $10^{-8}$	1531	2.0276 × $10^{-7}$
72	1409	2.3603 × $10^{-8}$	1391	5.9767 × $10^{-4}$

**Table 5:** Biochemical parameters in plasma in C57BL/6 mice treated chronically for 40 days with PsBu and APAP.

Plasma Metabolites	CONT	APAP	PsBu
Glucose (mg dL^−1^)	185.23 ± 12.03	233.08 ± 15.80 *	211.50 ± 14.04
Triglycerides (mg dL^−1^)	157.93 ± 11.76	138.85 ± 6.22	152. ± 17.79
Cholesterol (mg dL^−1^)	87.76 ± 5.97	104.78 ± 4.32	83.84 ± 21.68
HDL (mg dL^−1^)	60.71 ± 1.91	74.43 ± 6.33	52.02 ± 14.09
Uric acid (mg dL^−1^)	0.07 ± 0.01	0.06 ± 0.01	0.09 ± 0.03
Urea (mg dL^−1^)	33.44 ± 1.01	32.18 ± 1.54	40.26 ± 9.47
Creatinine (mg dL^−1^)	0.20 ± 0.02	0.21 ± 0.04	0.27 ± 0.11
Bilirubin (mg dL^−1^)	0.10 ± 0.02	0.24 ± 0.03 *	0.31 ± 0.07 **
AST (UI)	74.90 ± 4.46	65.50 ± 8.13	88.84 ± 11.75
ALT (UI)	23.10 ± 1.37	28.88 ± 5.26	30.78 ± 9.10

Biochemical metabolic parameters in the plasma of C57BL/6 mice treated chronically for 40 days with a daily oral dose (via gavage) of 200 mg kg^−1^ of polysaccharide extracted from *B. utriformis* (PsBu) and 300 mg kg^−1^ paracetamol (APAP), APAP alone, or those treated with vehicle (0.5% methylcellulose in 0.9% NaCl) as the control group (CONT). Values are presented as mean ± SEM (standard error of the mean) (*n* = 5–8 animals per group). Data were analyzed by one-way ANOVA for each parameter, followed by Bonferroni’s post hoc test for multiple comparisons. Significant differences compared with the CONT group are indicated as follows: (*) *p* < 0.05 and (**) *p* < 0.01. AST: aspartate aminotransferase; ALT: alanine aminotransferase.

## Data Availability

The original contributions presented in this study are included in the article/Supplementary Materials. Further inquiries can be directed to the corresponding author.

## Supplementary Materials

The following supporting information can be downloaded at: https://www.mdpi.com/article/10.3390/pharmaceutics17030335/s1, it includes details on ethical considerations and compliance with the ARRIVE Essential 10 guidelines for animal researches [99,100,101,102,103,104].
